# Cell Ferroptosis: New Mechanism and New Hope for Retinitis Pigmentosa

**DOI:** 10.3390/cells10082153

**Published:** 2021-08-21

**Authors:** Ming Yang, Kwok-Fai So, Wai-Ching Lam, Amy Cheuk Yin Lo

**Affiliations:** 1 Department of Ophthalmology, Li Ka Shing Faculty of Medicine, The University of Hong Kong, Hong Kong 999077, China; hrmeym@connect.hku.hk (M.Y.); hrmaskf@hku.hk (K.-F.S.); 2 State Key Laboratory of Brain and Cognitive Sciences, The University of Hong Kong, Hong Kong 999077, China; 3 GHM Institute of CNS Regeneration, Jinan University, Guangzhou 510632, China

**Keywords:** eye, homeostasis, ocular stress, regulated cell death, retina, vision

## Abstract

Retinitis pigmentosa (RP) is a leading cause of inherited retinal degeneration, with more than 60 gene mutations. Despite the genetic heterogenicity, photoreceptor cell damage remains the hallmark of RP pathology. As a result, RP patients usually suffer from reduced night vision, loss of peripheral vision, decreased visual acuity, and impaired color perception. Although photoreceptor cell death is the primary outcome of RP, the underlying mechanisms are not completely elucidated. Ferroptosis is a novel programmed cell death, with characteristic iron overload and lipid peroxidation. Recent studies, using in vitro and in vivo RP models, discovered the involvement of ferroptosis-associated cell death, suggesting a possible new mechanism for RP pathogenesis. In this review, we discuss the association between ferroptosis and photoreceptor cell damage, and its implication in the pathogenesis of RP. We propose that ferroptotic cell death not only opens up a new research area in RP, but may also serve as a novel therapeutic target for RP.

## 1. Introduction

Retinitis pigmentosa (RP) is the most common hereditary and non-nutritional retinal degenerative disease globally [1,2]. It is manifested by the chronic progressive loss of peripheral visual field and electrical potential abnormality, accompanied by reduced vision [2]. Most cases are inherited in an autosomal recessive, autosomal dominant, or X-linked inheritance manner [2,3].

Night blindness, progressive visual field deficiency, decreased central vision, and color vision abnormality, are the major symptoms of RP patients [1]. Night blindness is one of the first characteristic manifestations of RP. Patients may have difficulty seeing or walking at night or in dark places. In addition, an average of about 4.6% of the visual field is lost every year [4]. Most patients with onset at an early age have a typical progressive narrowing of the visual field, such as ‘’tubular visual field’’ [5]. The degree of decreased central vision is related to the stages of RP [6]. Although there is no color vision abnormality in the early stage of RP, color vision loss occurs when the visual acuity drops below 0.5 (Log MAR). The typical manifestation is blue blindness; red–green vision disorders are relatively rare.

RP is diagnosed by genetic testing, electroretinography (ERG), and visual field testing, as well as optical coherence tomography (OCT) [1,2,5,6]. Genetic testing enables identification of genes that are associated with RP, to evaluate the possibility of gene therapy. The ERG test provides data on the decreased responsiveness of retinal cells in RP patients. Visual field testing can help to identify peripheral vision loss in RP patients. Using OCT, images of various retinal layers are available, which can help to identify cytoarchitectural abnormalities, such as thinning of the photoreceptor layer [2,6]. Fundus examination could also highlight the “triad” of RP, i.e., optic nerve atrophy, narrowed retinal blood vessels, and bone spicules retinal pigmentation [6].

Although photoreceptor cell death is the primary outcome of RP, the underlying mechanisms are not completely elucidated [7]. Ferroptosis is a novel programmed cell death, with the characteristics of iron overload and lipid peroxidation [8,9]. Recent studies, using in vitro and in vivo RP models demonstrated features of ferroptosis in photoreceptor cells [10,11,12], suggesting possible new mechanism in RP pathogenesis. In this review, we discussed the association between ferroptosis and photoreceptor cell damage, and its implication in the pathogenesis of RP. Here, we propose that ferroptotic cell death not only opens up a new research area in RP, but may also serve as a novel therapeutic target for RP.

## 2. Overview of Ferroptosis

Death is the ultimate destination of all cells. In the 19th century, people found that cell death happened when a cell responded to unbearable pressure or damage, either by cell swelling or cell shrinkage; this was later classified into necrosis or apoptosis [13].

Ferroptosis is a new type of programmed cell death, named in 2012 [8]. It plays an important role in a wide range of diseases [9]. Whereas pyroptosis is inflammasome-mediated cell death [14,15,16], ferroptosis is a form of cell death that is caused by the accumulation of intracellular iron and lipid reactive oxygen species (L-ROS). Intracellular oxidation is the executer of ferroptosis, and is controlled by glutathione peroxidase 4 (GPx-4) [17] or ferroptosis suppressor protein 1 (FSP1) [18,19].

Ferroptosis can be inhibited by antioxidants and iron chelators, but it cannot be inhibited by apoptosis inhibitors [20]. Iron chelating agent, such as deferoxamine (DFO), reduces iron overload and lipid peroxides [21,22]. Ferroptosis is an iron-dependent cell death, characterized by iron overload, with an abnormal increase in lipid reactive oxygen species [23]. Transferrin is an iron-transporting protein, which recognizes and binds to transferrin receptor 1 (TFR1), locates on the cell membrane, to transfer ferric iron (Fe^3+^) from the extracellular environment to the cytoplasm [24]. Recently, TFR has also been considered as a specific ferroptosis marker, which stains ferroptotic cells [25]. STEAP3, a metalloreductase, transforms Fe^3+^ (insoluble) into Fe^2+^ (soluble), which is stored in the endosome [26]. When the cell suffers from stress, a large amount Fe^2+^ is released, through divalent metal transporter 1 (also called DMT1 or SLC11A2), into the cytoplasm, forming a labile iron pool [27]. The released Fe^2+^ could react with hydrogen peroxide, which is generated from the constant metabolism of the mitochondria, and result in the Fenton reaction, which is a reaction involving hydrogen peroxide and Fe^2+^ [27]. The Fenton reaction can be written (ferric iron and ferrous are both complexed to ligand ‘‘L’’) as L-Fe^2+^ + H_2_O_2_ → L-Fe^3+^ + OH^•^ + OH^−^. As a result, hydroxyl free radicals, such as OH, are generated, which leads to a multi-step free radical chain reaction, generating massive lipid-carrying reactive oxygen species (ROS) [28].

Polyunsaturated fatty acid (PUFA) is a major structural component of the cell membrane phospholipid, which is responsible for the fluidity of the cell membrane [29]. It is highly susceptible to oxidative stress and can easily be oxidized. Excessive PUFA oxidation subsequently generates peroxidized phospholipids, which drive ferroptosis [29]. Because of the continuous renewal of the cell membrane, newly made lipids are in constant need to supplement the lipid bilayer. As a result, oxidized PUFA readily enters the cell membrane, causing lipid peroxidation and cell membrane damage.

Glutathione peroxidase 4 (GPx-4) is the only enzyme that prevents destructive lipid peroxidation and catalyzes glutathione (GSH), and helps to remove lipid ROS [9]. However, due to the limited source of GSH and the expression of GPx-4, this protective mechanism could not handle massive lipid peroxidation, and, consequently, ferroptosis may happen. Inducers and inhibitors of ferroptosis facilitate the recent development of this subject (Table 1). Here, we review the following two classical pathways of ferroptosis: GSH/GPx-4 and FSP1/CoQ/NADPH.

### 2.1. GSH/GPx-4 Signaling

The peroxidation of polyunsaturated fatty acid (PUFA) in cells is catalyzed into polyunsaturated fatty acid-containing phospholipids (PL-PUFA (PE)) by acyl-CoA synthetase long-chain family member 4 (ACSL4), generating phospholipid hydroperoxide (PLOOH) [29]. PLOOH could trigger the iron-dependent self-amplified Fenton reaction, eventually leading to cell death if not inhibited [48]. However, GPx-4 can effectively reduce phospholipid peroxide and inhibit the production of arachidonic acid (AA), a long-chain fatty acid anion [17]. As GPx-4 is the only enzyme that is responsible for the reduction in PLOOH in mammalian cells, it plays an indispensable role in protecting cells from oxidative damage [49]. GSH is an essential cofactor for GPx-4; the consumption of GSH in the cell significantly reduces the activity of the GPx-4 enzyme and increases the intracellular lipid hydroxyl radicals. As a result, the accumulation of L-ROS and the oxidation of cell membrane damage lead to ferroptosis [9].

### 2.2. FSP1/CoQ/NADPH Signaling

In 2019, ferroptosis suppressor protein 1 (FSP1) was demonstrated to be a ferroptosis inhibitor, independent of the GSH/GPX4 signaling [18,19]. FSP1 was previously known as apoptosis-inducing factor mitochondria-associated 2 (AIFM2). However, due to the lack of an N-terminal mitochondrial targeting sequence in AIFM2, it is neither localized in the mitochondria nor is it a pro-apoptosis protein [19]. Therefore, AIFM2 was recently renamed as FSP1. Bersuker et al. and Doll et al. [18,19] found that FSP1 effectively inhibited ferroptosis-independent glutathione and GPx-4 signaling. It can function similarly to NADPH-dependent coenzyme Q (an oxidoreductase), which catalyzes the regeneration of CoQ_10_, using NADPH. Ubiquinol (CoQ_10_H_2_), the reduced form of CoQ_10_, traps free radicals that are generated by lipid peroxidation, thereby preventing membrane damage [18,19]. Further investigation of the FSP1/NADPH/CoQ_10_ pathway will be useful for understanding the biological mechanisms that regulate ferroptosis in different diseases.

### 2.3. Autophagy Regulates Ferroptosis

Autophagy is a “self-eating” process that is involved in multiple biological functions [50,51]. Correlations between autophagy and ferroptosis have been shown in recent studies. The activation of ferroptotic death depends on the induction of autophagy, and ferroptosis regulatory proteins may also be involved in the regulation of autophagy [52,53].

Cells treated with ferroptosis inducers, such as erastin, increased autophagy, whereas autophagy-deficient cells showed higher cell viability [53]. Excessive activation of intracellular autophagy or lysosomal activity leads to the accumulation of intracellular free iron and lipid peroxides, thereby promoting the occurrence of ferroptosis [54]. In general, autophagy regulates ferroptosis through the following four pathways: ferritinophagy, lipophagy, clockophagy, and chaperone-mediated autophagy.

**Figure 1:**
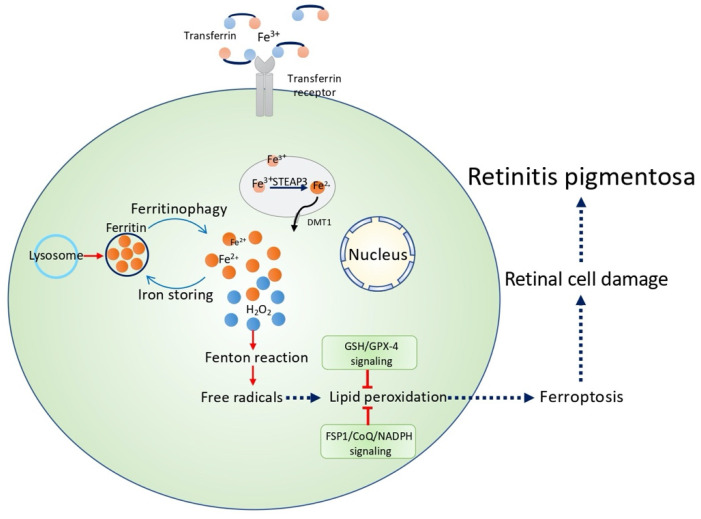
A schematic figure showing ferroptosis as the potential mechanism for retinitis pigmentosa through GSH/GPx-4 signaling or FSP1/CoQ/NADPH signaling.

Ferritinophagy is a prerequisite for ferroptosis [55,56,57]. Under normal physiological conditions, excessive Fe^2+^ in the cell is oxidized to Fe^3+^ by the ferritin heavy-chain subunit (FTH) and, in turn, stored in ferritin or transported out of the cell by the iron transport export protein 1 (ferroprotein 1, FPN1) [9]. It has been shown that nuclear receptor coactivator 4 (NCOA4) interacts with FTH to mediate the autophagic degradation of ferritin [56]. This process is called ferritin autophagy (ferritinophagy). The autophagic degradation of ferritin promotes the release of Fe^3+^, which causes the increase in free iron in the cells, and thus promotes ferroptosis [58]. The knockdown or knockout of NCOA4, and inhibition the of autophagy, could effectively prevent the occurrence of ferroptosis [58]. Ferritinophagy is required for the induction of ferroptosis, but ferroptosis inducers can promote ferritinophagy as well [55,59] (Figure 1).

“Lipophagy” is the degradation process of intracellular lipid droplets, through autophagy [60]. Free fatty acids, generated by lipid autophagy, promote ATP production through β-oxidation in the mitochondria. Current studies have shown that lipophagy promotes RSL3-induced lipid peroxidation and ferroptosis, and the overexpression of tumor protein D52 promotes lipid storage, while inhibiting lipophagy, which effectively inhibits RSL3-induced lipid peroxidation and ferroptosis [61]. However, the potential involvement of other lipid autophagy regulatory proteins in the regulation of ferroptosis is still not fully elucidated.

Clockophagy is a newly discovered autophagy process in 2019 [62]. The circadian clock is endogenous, and can regulate the conversion of circadian rhythm and control many cellular physiological processes, including iron metabolism, oxidative stress, and cell death [63]. Studies have shown that the core protein of the biological clock, aryl hydrocarbon receptor nuclear translocation-like protein 1 (ARNTL1), can be degraded by autophagy, and this process is mediated by p62. This leads to increased expression of hypoxia-inducible factor prolyl hydroxylase 1 (PHD1), thereby promoting intracellular lipid peroxidation and further enhancing ferroptosis [62,63]. As clockophagy happens when there is excessive or impaired autophagy, it is not a normal process.

Chaperone-mediated autophagy (CMA) first entails the recognition of the special amino acid sequence ‘‘KFERQ’’ in the substrate, by the molecular chaperone, which then enters the lysosome by binding to lysosome-associated membrane protein type 2A (LAMP2A), thereby degrading the substrate [64,65]. Besides removing lipid peroxides in the cell, to protect it from ferroptosis, GPx-4 also interacts with the heat shock protein family A member 8 (HSPA8), which is a molecular chaperone that is degraded by CMA. The overexpression of LAMP2A promotes CMA to degrade GPx-4, leading to ferroptosis [66,67,68]. However, contrary to HSPA8, HSPA5 prevents the degradation of GPx-4, by interacting with GPx-4, thereby preventing ferroptosis [67]. The regulatory proteins or factors that are involved in regulating the process of ferroptosis, are also implicated in controlling autophagy (Table 2). Future drugs may be designed for these common regulatory factors, which, at the same time, intervene with autophagy and ferroptosis, to treat RP. However, when designing drugs for these co-regulatory proteins, it is necessary to comprehensively consider the changes in autophagy and ferroptosis in the disease. In addition, how these common regulatory proteins regulate autophagy or ferroptosis, or both, needs further study.

## 3. Disease Mechanisms of RP

### 3.1. Pathophysiology

RP is a retinal degenerative disease in which photoreceptor degeneration (mostly rods and later cones). It is also coined photoreceptor dystrophy [6]. The degeneration originates from the peripheral retina, and progresses to the macula and fovea [70], constricting the visual field [7]. As a result, RP patients develop symptoms such as night blindness, reduced visual field, and decreased vision [71,72]. Abnormal choroidal circulation may also contribute to RP. It is possible to use an electroretinogram (F-ERG), electrooculogram (EOG), and visual evoked potential (P-VEP), to objectively determine whether the choroidal circulation abnormalities affect the retina, macula, or optic nerve [1,6].

### 3.2. Genetic Mechanisms

RP is a genetic disease [6]. There are three types of inheritance: autosomal recessive, dominant, and sex-linked recessive [73]. The autosomal recessive inheritance is the most prevalent, followed by the dominant and the sex-linked recessive inheritance [74]. Currently, it is believed that the autosomal dominant genetic type has at least 17 gene loci located on the short arm of chromosome 1 and the long arm of chromosome 3 [75]. Sex-linked genetic genes are located in the first and second regions of the short arm of the X chromosome [3,76].

### 3.3. Other Molecular Mechanisms

In recent years, studies found that RP patients have abnormalities in humoral and cellular immunity [77]. Compared with healthy subjects, RP patients carry activated T cells, B cells, and macrophages in the vitreous, while their RPE cells express HLA-DR antigens [78]. In addition, it has been reported that some patients have autoimmunity problems [79], but evidence is insufficient to support this claim. Blood biochemical analysis in RP patients also showed the presence of abnormal lipid metabolism, aberrant trace elements (zinc, copper, selenium) and enzyme metabolism, together with lipofuscin accumulation in the retina [6].

Autophagy has been considered to be a pathological mechanism of RP. It was shown that light-induced damage promoted the expression of visual cytotoxic signaling proteins, leading to retinal degeneration [80]. The knockout of the autophagy gene Agt5 aggravated the accumulation of these proteins, thereby accelerating retinal degeneration. The double knockout of Agt5 and visual transduction protein gene Gnat-1 could lead to a decreased rate of rod cell degeneration, with a well-preserved photoreceptor. Therefore, autophagy may help to clear the visual transduction protein in time, to protect photoreceptors. The Epg5 gene is a unique and highly conserved autophagy gene in multicellular organisms. Epg5 knockout mice also showed progressive retinal neurodegenerative changes in the retina. During the aging process of Epg5 knockout mice, retinal photoreceptor cells gradually degenerate, which is accompanied by progressive visual dysfunction [81]. Also, the apoptotic activity and the number of apoptotic cells in the ONL of the Epg5 knockout mouse retina, are significantly increased compared with the control group. Therefore, Epg5-deficient mice displays features of RP. These results further support the association of autophagy in the pathogenesis of RP.

## 4. Prevention and Intervention Strategies of RP

RP is characterized by the irreversible loss of photoreceptor cells, accompanied by RPE cells, and there is no effective treatment for RP yet [82]. For people with impaired vision, a typoscope can be worn to improve their vision for reading. Meanwhile, RPE [83], retinal photoreceptor cell [84], retinal progenitor cell [85], and iris pigment epithelial cell (IPE) [86], have been selected as candidates for cell transplantation, with varying results. Some studies have successfully established the cultivation of photoreceptor cells. While the technique of the sheet transplantation of allogenic photoreceptor cells in animals is successful, there are still doubts about the functional reconstruction [87,88,89,90]. Allogenic RPE transplantation can induce the immunological rejection reaction [91], but it is difficult to collect autologous RPE. Therefore, the possibility of replacing RPE with iris pigment epithelial cell (IPE) has been considered and discussed [86,92]. However, this treatment is only for RPE disease, while RP is overwhelmingly a disease of photoreceptor damage. Induced pluripotent stem cell (iPSC) can differentiate to RPE and photoreceptor cells. Transplantation of these derived cells has been shown to rescue light perception and visual function in RCS rats and NOD.SCID-rd1 mice [83]. Another study also demonstrated that the long-term treatment of iPSC-derived RPE and photoreceptor cells effectively protected Pde6b knockout rats [93]. These findings suggest that iPSCs may be a promising therapeutic method for RP.

### 4.1. Gene Therapy

RP has typical genetic heterogeneity. The various pathogenic genes that have been identified, have different inheritance patterns and different gene abnormalities [76,94]. Although the FDA has approved several gene therapy drugs, the presence of a plethora of genes involved in RP, and the difficulty in identifying exact gene mutations, limit the type of gene therapy. On the other hand, RPE65 bi-allelic mutations cause Leber congenital amaurosis (LCA), which can also lead to RP [95]. Subretinal injection of adeno-associated virus (AAV) vector, inserted with RPE65 cDNA, repairs this genetic deficiency. In 2017, a phase III clinical trial demonstrated that voretigene neparvovec (AAV2-hRPE65v2) gene therapy showed effective treatment outcomes in 31 patients with RPE 65-mediated inherited retinal dystrophy (ClinicalTrials.gov number, NCT00999609) [96]. This shows that gene therapy is a viable option for some cases of RP.

Meanwhile, hereditary RP is characterized by cell apoptosis, which can be targeted [7]. A first-in-human gene treatment has reported promising safety and efficacy on RPGR-mutated X-linked RP, with improved visual acuity and microperimetry [97]. Studies have reported that the inhibition of apoptotic genes and various growth factors, such as brain-derived neurotrophic factor (BDNF) [98], basic fibroblast growth factor (bFGF) [99], ciliary neurotrophic factor (CNTF) [100], and glial cell-derived growth factor (GDNF) [101], could delay photoreceptor cell apoptosis. However, the key factors in the pathogenesis and treatment of RP remain to be elucidated.

### 4.2. Drug Treatment

Vitamin A, taurine, and docosahexaenoic acid (DHA) are the most commonly used regimens for abnormal biochemical metabolism in RP patients [102]. They all participate in the maintenance of the normal structure and function of RPE and photoreceptor cells, as well as the normal photoelectric conversion reaction. However, it has been reported that a high dose of vitamin A treatment increased the risk of visual loss, making the value of this type of treatment doubtful [103].

Neurotrophic factors are important for the survival, development, and programmed death of neurons. RPE and Mǜller cells release neurotrophic factors, such as CNTF, FGF, transforming growth factor-α, -β (TGF-α, -β), and platelet-derived growth factor (PDGF), into the intercellular space among photoreceptor cells [104]. Although the concentrations of these factors are low, they induce strong beneficial biological effects, such as cell death prevention and cell growth promotion, rescuing photoreceptors degeneration, and restoring RPE phagocytic function that can help treat RP. Therefore, delivering neurotrophic factors into photoreceptor cells, while minimizing the damage to other tissues of the eye, may be a potential therapy. However, current research on the neurotrophic factors for the treatment of RP is still limited to animal studies.

### 4.3. Others

As strong light accelerates the degeneration of the outer segment of the photoreceptors, sunglasses are important for RP patients. Surgeries using autologous blood vessels to create collaterals, can increase the blood supply to the fundus, which can help photoreceptors in their malnutrition stage, to restore their functions [71]. A healthy lifestyle, such as regular sleeping, not smoking, and a healthy diet, is also essential. Although it is unlikely to accurately predict the individual development of RP, frequent monitoring of RP, by electroretinogram and visual field testing, helps to achieve timely control, preventing further retinal degeneration for early and mid-stage RP patients [105]. Concomitant ophthalmic diseases, such as cataract and cystoid macular edema, which may associate with RP, should also be prevented [106]. For advanced patients, preventing the progression of the disease, and the replacement of degenerated photoreceptor cells or a retina implant, may offer partial sight restoration [107].

To conclude, effective treatment for RP is still lacking. Oral vitamin A, vitamin E, DHA, or neuroprotective factors, may delay photoreceptor cell death, thereby ameliorating the progression of the disease. However, there are controversies regarding the specific efficacy of these drugs.

## 5. Association between Ferroptosis and RP

In spite of the recent signs of progress in ferroptosis study, no systematic reports associating RP and ferroptosis are available yet. However, RP has displayed several features that are related to ferroptosis, such as the expression of GPx-4 and GSH, as well as lipid peroxidation (Table 3).

### 5.1. Potential Involvement of Ferroptosis in RP

Genetic mutations trigger rod cell death. As a result, oxygen consumption ability is decreased in the outer retina. Together with the redundant oxygen generating oxidative stress, including hydrogen peroxide, this could also cause accumulative oxidative damage to cones, leading to cell death. On the other hand, iron metabolic dysfunction, causing iron accumulation or iron overload, has been reported in RP models [10,11]. A study found the increase levels of both transferrin and transferrin receptors was accompanied by an increased ferritin and ferritin-bound iron level in *rd10* mice [11]. Moreover, the level of 4-hydroxy-2-nonenal (4-HNE), a marker of lipid peroxidation, is also significantly increased in the retina [11]. These results suggested the potential involvement of ferroptosis in *rd10* mice. However, a link between iron overload and lipid peroxidation is still lacking. A recent study shows that the intravitreal injection of Fe^2+^ significantly induced photoreceptor oxidative stress and degeneration, while increasing 4-HNE and decreasing GPx-4 level [12]. Based on the theory of ferroptosis, excessive cytoplasm-free iron, together with lipids and hydrogen peroxide, results in the Fenton reaction, producing massive lipid ROS, causing lipid peroxidation. This evidence supports a possible involvement of ferroptosis in RP.

### 5.2. Ferroptosis Regulation in RP

Increasing evidence, showing the regulation of ferroptosis, has a great potential for RP treatment. In 2009, there was a breakthrough study, showing that the overexpression of GPX-4 strongly protected the retina in three models of oxidative damage-induced retinal degeneration [108]. Moreover, iron chelators, well-recognized ferroptosis inhibitors, show protective effects in several RP models. In 2011, a study demonstrated that zinc-desferrioxamine significantly alleviated retinal degeneration in *rd10* mice [112]. Also, in the same RP model, two other iron-chelating drugs, including VK28 and VAR10303, were shown to promote cone photoreceptor survival, suggesting that chelating labile iron is a novel treatment option for RP [113]. In 2013, a study also illustrated that an iron chelator deferiprone decreased oxidative stress, thereby protecting the degeneration of the light-exposed photoreceptor in mice [109]. These studies together suggest the potential of iron regulation in RP treatment. However, data on other ferroptosis regulators, such as ferrostatin-1 and liproxstatin-1 on RP models, are still lacking. Recently, two new studies found that ferroptosis is involved in sodium iodate-induced ARPE-19 cell death, suggesting that ferroptosis is involved in retinal degeneration, which supports the association with RP [110,111]. They first observed that sodium iodate increased intracellular labile iron, while decreasing intracellular glutathione and cysteine. Using deferoxamine or ferrostatin-1, two ferroptosis inhibitors, ARPE-19 cell death was prevented from sodium iodate exposure [110,111]. These studies together support the relatively strong association between RP and ferroptosis (Figure 1). However, further studies on ferroptosis are essential.

## 6. Ferroptosis: A New Future Therapeutic Target for RP?

Although gene therapy, drug treatment, and iPSCs are available for RP, they might still have some disadvantages. Current therapeutic options are nonetheless limited. Therefore, it is important to seek alternative choices, by uncovering novel mechanisms of RP. Ferroptosis is closely associated with RP pathogenesis. Therefore, it would be worthwhile to investigate whether anti-ferroptotic events, such as decreasing iron overload, increasing the level of anti-oxidants, and reducing lipid peroxidation, could rescue photoreceptors in RP. Apart from using iron chelators, increasing the expressions of GSH and GPX-4, while decreasing ACSL-4, could be helpful to further evaluate the role of ferroptosis in RP pathogenesis.

As shown in the previous section, ferroptosis is regulated by autophagy in some diseases, such as cancer and neurodegeneration [114]. Evidence also supports the important role of autophagy in maintaining photoreceptor homeostasis. Therefore, it may be possible to control ferroptosis in RP by regulating autophagy.

## 7. Conclusions and Future Remarks

RP is a hereditary eye disease. The current FDA-approved gene therapies are expensive. They are effective in young patients, but a personalized regimen is not feasible, due to the large genetic variations in RP. There is still a lack of good therapeutic strategies that effectively delay the progression of RP and preserve the patients’ vision. Numerous attempts have been tried, to find neuroprotective drugs (neurotrophic factors, anti-oxidants, and anti-apoptotic factors), but they all have limitations and complications.

In summary, the search for novel mechanisms of photoreceptor cell death, such as ferroptosis, may contribute to our understanding of RP and provide new directions for its treatment.

## Figures and Tables

**Table 1:** A summary of molecules for ferroptosis regulation.

	Classification by Mechanisms	Name	Molecular Weight	References
**Inducers**	Inhibition of system X_c_^−^	Erastins	547.04	[30]
		Sorafenib	464.82	[31]
		Sulfasalazine	398.39	[32]
	Inhibition of cystine consumption	Glutamate	365.34	[33]
	Inhibition of GPx-4	Altretamine	210.28	[34]
		(1S, 3R)-RSL3	440.88	[17]
	Depletion of GSH	Cisplatin	300.05	[35]
		Artesunate	384.42	[36]
		DPI2	314.55	[37]
		Buthionine sulfoximine	222.31	[38]
		Ferrous chloride	537.26	[39]
		Ferrous ammonium sulfate	392.14	[40]
		Ferric ammonium citrate	Variable	[41]
**Inhibitors**	Anti-lipid peroxidation	Ferrostatin-1	262.35	[42]
		Liproxstatin-1	340.85	[43]
		Vitamin E	430.71	[44]
		SRS 11–92	352.47	[27]
		SRS 16–86	432.6	[45]
		CoQ_10_	863.34	[18]
		Idebenone	338.439	[46]
	Reducing iron overload	Deferoxamine	560.484	[47]
		Deferiprone	139.152	[23]
		Ciclopirox	268.35	[37]

System Xc^−^: the cystine/glutamate transporter/antiporter system; GPx-4: glutathione peroxidase 4; GSH: glutathione; CoQ_10_: coenzyme Q_10_.

**Table 2:** Pathways of ferroptosis regulation by autophagy.

Type	Mediator	Effect	References
Ferritinophagy	NCOA4	Lipid peroxidation Ferroptosis	[54]
Lipophagy	RSL3		[69]
Clockophagy	P62, ARNTL, PHD1		[62]
CMA	HSPA8, LAMP2A, GPX-4		[67]

**Table 3:** Evidence supporting the association between retinitis pigmentosa and ferroptosis.

Subject	Effect on Iron Homeostasis	Effect on Ferroptosis-Associated Protein Expression	Effect on Oxidative Stress	Effect on Lipid Peroxidation	Effect on Retina/Cell/Mitochondria Morphology	References
Oxidative damage-induced retinal degeneration	N/A	Decreased GPX4 expression	Decreased ROS level and increased SOD1 expression	N/A	Improved retina thickness and cell loss	[108]
*rd10* mice	Increased expressions of transferrin, ferritin, ferritin-bound iron, ceruloplasmin and total retinal iron	Decreased GPX expression	N/A	Increased HNE level	N/A	[11]
Intravitreal injection of ferrous on wild type and *rd10* mice	N/A	Decreased GPX expression	Increased Hmox1, SOD1 and SOD2 and oxidative damage to the retina	Increased HNE level	Increased pan-retinal autofluorescence and photoreceptor degeneration	[12]
Light-induced retinal degeneration	Increased ceruloplasmin	N/A	Increased oxidative stress	N/A	Increased retinal structure damage	[109]
Sodium iodate-induced ARPE-19 damage	Increased labile iron	N/A	Increased oxidative stress	Increased lipid peroxidation	N/A	[110]
Sodium iodate-induced retinal degeneration	Increased labile iron	Decreased GPX-4 expression	Increased oxidative stress	Increased lipid peroxidation	Increased retinal structure damage	[111]

## Data Availability

Not applicable.
